# Transition Pattern and Mechanism of B-lymphocyte Precursors in Regenerated Mouse Bone Marrow after Subtotal Body Irradiation

**DOI:** 10.1371/journal.pone.0046560

**Published:** 2012-10-17

**Authors:** Deping Han, Mei Zhang, Jun Ma, Jingshen Hong, Chun Chen, Bingrong Zhang, Luqiang Huang, Wenlong Lv, Liangjie Yin, Amy Zhang, Hengshan Zhang, Zhenhuan Zhang, Sadasivan Vidyasagar, Paul Okunieff, Lurong Zhang

**Affiliations:** 1 First Affiliated Hospital, Fujian Medical University, Fuzhou, Fujian, China; 2 Department of Radiation Oncology, UF Shands Cancer Center, Gainesville, Florida, United States of America; 3 Institute of Digestive Diseases, Zhengzhou University, Henan, China; Children's Hospital Boston, United States of America

## Abstract

Little is known about the effects of ionizing radiation on the transition and the related signal transduction of progenitor B cells in the bone marrow. Thus, using an NIH Swiss mouse model, we explored the impact of ionizing radiation on the early stage of B-cell development via an examination of the transition of CLP to pro-B to pre-B cells within bone marrow as a function of radiation doses and times. Our results showed that while the total number of bone marrow lymphoid cells at different stages were greatly reduced by subtotal body irradiation (sub-TBI), the surviving cells continued to transition from common lymphoid progenitors to pro-B and then to pre-B in a reproducible temporal pattern. The rearrangement of the immunoglobulin heavy chain increased significantly 1–2 weeks after irradiation, but no change occurred after 3–4 weeks. The rearrangement of the immunoglobulin light chain decreased significantly 1–2 weeks after sub-TBI but increased dramatically after 3–4 weeks. In addition, several key transcription factors and signaling pathways were involved in B-precursor transitions after sub-TBI. The data indicate that week 2 after irradiation is a critical time for the transition from pro-B cells to pre-B cells, reflecting that the functional processes for different B-cell stages are well preserved even after high-dose irradiation.

## Introduction

Bone marrow is the primary tissue that produces hematopoietic stem cells and hosts some transitions from stem cells to differentiated cells, including precursors at different stages in the development of B lymphocytes. While the stem cells or precursors in bone marrow are extremely vulnerable to radiation, as evidenced by a sharp decline in the number of precursors or stem cells and consequently the number of lymphocytes, neutrophils, and platelets in the peripheral blood [1], [2], the bone marrow possesses a powerful repair and regenerative capacity that compensates for the lost cells and maintains homeostasis [3], [4].

Because a sufficient number of lymphocytes are needed for adequate immunosurveillance, maintaining the primary lymphocyte pools at different stages is crucial. Radiation-induced immunosuppression leads to the emergence of opportunistic infections that may be lethal, depending on the radiation dose, dose rate, and duration of exposure [5]. Thus, the protection of immunocompetent cells from radiation damage is essential for their survival. Among these cells, B lymphocytes play a major role in the humoral immune response. B-cell precursors are among the best-characterized hematopoietic precursors, and extensive study of their development has helped to determine potential pathways for the advancement of various blood-cell lineages.

While B-lymphocyte precursors are ultrasensitive to radiation-induced DNA damage [6], they also regenerate and differentiate quickly in a preprogrammed fashion. The earliest B lineage–restricted precursors in adult bone marrow derive from common lymphoid progenitors (CLP), such as Lin^−^ IL-7R^+^ c-*kit* ^low^ Sca-1^low^ [7], which are limited in their ability to develop into T lymphocytes but are fully capable of developing into B cells. B-cell development is divided into several successive steps, as judged by surface markers and the rearrangement of immunoglobulin (Ig) genes [8]. The first B lineage-restricted cells are termed pro-B cells (defined as c-Kit^+^ CD19^+^ IgM^−^ cells), which initiates rearrangement at the Ig heavy chain (IgH) locus: D_H_ to J_H_ joining at the early pro-B cell stage, followed by V_H_ to DJ_H_ joining at the late pro-B cell stage [9], [10]. Those pro-B cells that successfully undergo V(D)J recombination of the IgH locus proceed to the pre-B cell (defined as CD25^+^ CD19^+^ IgM^−^ cells) stage and express the pre-B cell receptor (pre-BCR), which consists of the productively rearranged IgH protein and the surrogate light chain (SLC), λ5, and VpreB, to mediate expansion of pre-B cells. Signaling through the pre-BCR results in a proliferation phase and initiates Ig light chain (IgL) recombination. When the SLC is replaced by a successfully rearranged IgL to form IgM, the cells become immature B cells [11]. This transition from pro-B cell to pre-B cell represents the first stage that directs B-cell development [12].

By destroying B cells or by some other mechanism, subtotal body irradiation (sub-TBI) stimulates residual bone marrow cells to proliferate and repopulate the lymphoid system [3], [13], leading to the restoration of B cells and antibody production [14], [15]. To better understand the radiobiology of bone marrow, we explored the transition of B precursors as a function of times and radiation doses and their related signal transductions during the recovery process.

## Materials and Methods

### Animal ethics

This study was performed in strict accordance with University of Florida Institute Animal Protocol. All efforts were made to minimize animal suffering and limit animal numbers. Any animals that showed signs of weakness and dehydration due to irradiation received a wet food diet. Animals that continued to exhibit signs of weakness were humanely euthanized through cervical dislocation. This protocol and all care and use procedures were approved by the Institutional Animal Care and Use Committee (IACUC) at the University of Florida.

### Animal irradiation and bone marrow cell preparation

Male NIH Swiss mice (6–8 weeks old) were purchased from the National Cancer Institute (NCI, Frederick, MD) and kept under specific pathogen-free conditions. Animals were divided into groups (*n* = 5 mice/group), maintained on a 12-hour light schedule, and fed a standard diet.

Mice were immobilized with plastic restrainers and subjected to 0, 5, or 10-Gy sub-TBI via a GammaCell 40 Irradiator with a cesium-137 source at a dose rate of 1 Gy/minute (Atomic Energy of Canada Limited Company, Chalk River, Ontario); the right hind leg was secured outside of the radiation field. Control mice were immobilized and sham irradiated.

At 1–4 weeks after irradiation, bone marrow cells were isolated from mouse femurs from both legs, as described by Nadri *et al.* [16]. Briefly, mice were euthanized by cervical dislocation, and their femurs were cleaned. The tip of each femur was removed, and the marrow was harvested by inserting a syringe needle (27 gauge) into one end of the bone and by flushing with Dulbecco's Modified Eagle's Medium (DMEM; Gibco Inc., Life Technologies, Grand Island, NY). The bone marrow cells were filtered through a 60-µm nylon mesh filter (Falcon, BD Biosciences, Franklin Lakes, NJ).

### Reagents

The antibodies used for flow cytometry analysis and Western blotting were purchased from Biolegend (San Diego, CA): anti-mouse lineage (2H7), c-kit (104D2), IL-7R (A7R34), IgM (RMM-1), CD19 (6D5), and CD25 (PC61); and from Cell Signaling Technology (Danvers, MA): anti-Stat 3 (124H6), anti-Stat 5 (4H1), anti-Akt (5G3), anti-Erk 1/2 (137F5), and anti-β-actin (13E5). The anti-p38 (137F5) was purchased from Sigma-Aldrich (St. Louis, MO).

### Flow cytometry analysis

Single-cell suspensions prepared from bone marrow were kept on ice and initially incubated with 10% newborn calf serum (NCS) in DMEM to block nonspecific antibody binding. To define the development stage of B precursors, different antibodies conjugated with fluorochrome against various differential markers were used to stain the bone marrow cells at 4°C for 30–45 minutes. After being washed twice with cold phosphate buffered saline (PBS), the stained cells were analyzed with an Accuri C6 Flow Cytometer (Ann Arbor, MI). CLP cells were defined with Lin^−^ C-kit^+^IL-7R^+^ IgM^−^ cells; pro-B cells with CD19^+^ IgM^−^ C-kit^+^; and pre-B cells with CD19^+^ IgM^−^ CD25^+^.

### Real-time polymerase chain reaction analysis

To determine B-precursor cell transition patterns, IgH and IgL gene rearrangements were also analyzed in bone marrow cells. Sequential genes targeting IgH and IgL rearrangements were applied in the following order: the first one targeted IgH proximal gene (V_H_7183-DJ) rearrangements; the second one targeted IgH distal gene (V_H_J558-DJ) rearrangements; the third one targeted SLC λ5 expression; the fourth targeted IgL κ gene (Vκ1-JCκ) rearrangement; and the final one targeted IgL λ gene (Vλ1-JCλ1) rearrangement. IgH gene (V_H_7183-DJ and V_H_J558-DJ) rearrangement was in the pro-B cell stage, and the IgLκ gene (Vκ1-JCκ) and λ gene (Vλ1-JCλ1) rearrangements were in the pre-B cell stage, whereas SLC λ5 expression was in the transition from the pro-B stage to the pre-B stage.

Expressions of the recombination activating gene (RAG) and the 3 transcription factors (E2A, EBF, and Pax5) that regulate Ig gene rearrangements were analyzed with real-time polymerase chain reaction (RT-PCR). Briefly, total RNA from bone marrow cells was extracted with a Qiagen kit (Valencia, CA). Thereafter, cDNA was synthesized with random hexamers and Superscript II reverse transcriptase (Invitrogen, Life Technologies). A Light Cycler DNA Master SYBR Green 1 kit (Applied Science, South Kingstown, RI) and quantitative PCR primers (Table 1) were used for RT-PCR analysis. A Bio-Rad I-cycler iQ RT-PCR detection system was employed to run the reactions. PCR assays were performed in duplicate or triplicate for each sample. The cycle number (Ct) at which the fluorescent signal of a given reaction crossed the threshold value was used as a basis for quantification. The difference in Ct (Δ Ct) was determined for comparison of the expression levels of indicated genes and transcription factors between groups.

**Table 1:** qPCR primers.

Rag1	5′ TGCAGACATTCTAGCACTCTGGCC 3′
	5′ ACATCTGCCTTCACGTCGATCCGG 3′
Pax5	5′ CCATCAGGACAGGACATGGAG 3′
	5′ GGCAAGTTCCACTATCCTTTGG 3′
E2A	5′ AGTGACCTCCTGGACTTCAG 3′
	5′ TGATCCGGGGAGTAGATCGA 3′
Ebfl	5′ GCATCCAACGGAGTGGAAG 3′
	5′ GATTTCCGCAGGTTAGAAGGC 3′
V_H_ 7183-DJCμ	5′ CGGTACCAAGAACAACCTGTACCTGCAAATGAGC 3′
	5′ ATGCAGATCTCTGTTTTTGCCTCC 3′
V_H_ J558-DJCμ	5′ CGAGCTCTCCAACACAGCCTACATGCAGCTCAAC 3′
	5′ ATGCAGATCTCTGTTTTTGCCTCC 3′
Vκ-JCκ	5′ GGCTGCAGCTTCAGTGGCAGTGGATCTGGAAC 3′
	5′ TTGGTCAACGTGAGGGTGCTG 3′
V_λ_1-JC_λ_	5′ CGCGAATTCTCAGGCTCCCTGATTGGAGACAAGG 3′
	5′ GACCTAGGAACAGTCAGCACGGG 3′
λ5	5′ GCGAAGCTTACACACTACGTGTGGCCTTGT 3′
	5′ GCGGAATTCTCAGCAGAAAGGAGCAGAGCT 3′
β-actin	5′ TACCACTGGCATCGTGATGGACT 3′
	5′ TTTCTGCATCCTGTCGGCAAT 3′

### Western blot

Bone marrow cells were washed with PBS, and the total protein was extracted with a lysis buffer [20 mmol/L HEPES (pH, 7.5), 10 mmol/L KCl, 1.5 mmol/L MgCl_2_, 1 mmol/L EDTA, 1 mmol/L EGTA, 1 mmol/L DTT, 250 mmol/L sucrose, 1 mmol/L phenylmethylsulfonyl fluoride, 1 mg/mL aprotinin, and 1 mg/mL pepstatin A] for 20 minutes at 4°C and then homogenized with sonication for 10 seconds. Cells were centrifuged at 10,000 rpm for 15 minutes at 4°C to extract the cytosol protein. The cytosol protein was subjected to 10% sodium dodecyl sulfate polyacrylamide gel electrophoresis (SDS-PAGE), transferred to an immunoblotting membrane, stained with monoclonal antibody, followed by secondary antibody conjugated to horseradish peroxidase (HRP), and then visualized with enhanced chemiluminescence (Pierce ECL, Thermo Fisher Scientific, Inc., Rockford, IL). For reprobing β-actin, the blots were stripped with a buffer containing 50 mmol/L Tris-HCl (pH, 6.8), 2% SDS, and 0.1 mol/L β-mercaptoethanol. The bands were scanned and analyzed with ImageJ software for density comparison.

### Statistical analysis

All data were expressed as the mean ± SD of at least 3 independent analyses. The differences between 2 groups were determined by the two-sample Student's *t*-test or one-factor analysis of variance.

## Results

### The effect of radiation on bone marrow cell count

**Figure 1:**
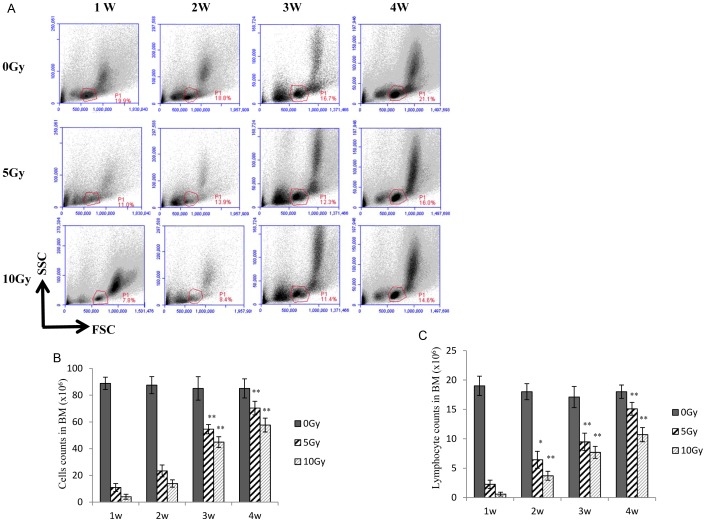
The effect of sub-TBI on mouse bone marrow cells. Flow cytometry analysis (**A**) was performed for the total bone marrow cells or lymphocytes in bone marrow as a function of sub-total body irradiation (sub-TBI) dose (0, 5, and 10 Gy) and time (1, 2, 3, and 4 weeks). The absolute numbers of total bone marrow cells (**B**) and total lymphocytes (**C**) were determined in the bone marrow isolated from the femur of the 2 hind legs of 6 to 8-week-old NIH Swiss mice (5/group) at 1, 2, 3, and 4 weeks after sub-TBI. Numbers represent the percentage of bone marrow cells in test mice, as compared to that of the control mice. Results are representative of 3 independent experiments. * represents *P*<0.05 and ** represents *P*<0.01, as compared with the 0-Gy group (*n* = 5).

Using the physical parameters of flow cytometry (forward scatter and side scatter), we studied radiation-induced damage on total bone marrow cells and lymphocytes as a function of doses and times. We also examined the regeneration pattern for these cells. Figure 1A shows that cells in the “lymphocyte area” were dramatically reduced to the nadir in the first week after irradiation, further decreased in the second week, but then increased on the third and fourth weeks; however, levels were still lower than those of the nonirradiated controls. The number of total bone marrow cells and lymphocytes in the bone marrow was inversely correlated with the radiation dose (Fig. 1B and 1C). At 2–4 weeks, the bone marrow cell and lymphocyte counts increased significantly in both the 5-Gy and 10-Gy groups, as compared to one week after irradiation. Since there was no antibody staining and the bone marrow was filled with peripheral blood, the cells in the “lymphocyte area” were likely mature monocytes/lymphocytes in the bone marrow. These results were consistent with previous reports [1], [3], [13].

### The effect of radiation on the transition from common lymphoid progenitor to pro-B to pre-B cells in the bone marrow

**Figure 2:**
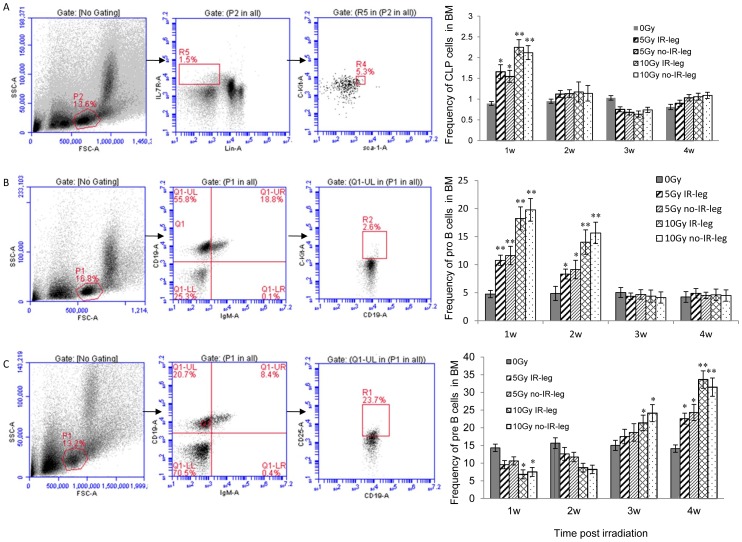
The effect of sub-TBI on the frequency of B precursors in mouse bone marrow. Flow cytometry analysis: (**A**) represents the percentage of the common lymphoid progenitor cells (CLP, Lin^−^ IL-7R^+^ c-*kit* ^low^ Sca-1^low^); (**B**) represents the pro-B cells (c-Kit^+^ CD19^+^ IgM^−^); and (**C**) represents pre-B cells (CD25^+^ CD19^+^ IgM^−^) in mouse bone marrow isolated from the femur of the 2 hind legs separately at 1, 2, 3, and 4 weeks after 5-Gy or 10-Gy sub-TBI. Results are representative of 3 independent experiments. * represents *P*<0.05 and ** represents *P*<0.01, as compared with the 0-Gy group (*n* = 5).

Bone marrow cells were isolated from the irradiated right leg and the nonirradiated left leg. Harvested cells were stained with a combination of monoclonal antibodies conjugated with fluorescence for different stages of progenitor cells. Compared to the control group, the 5-Gy and 10-Gy groups exhibited an increase in the percentage of CLP cells (Lin- C-kit^+^IL-7R^+^ IgM^−^) in bone marrow 1 week after irradiation but a significant decrease 3 weeks after irradiation (Fig. 2A and 2B). The change in CLP percentage was identical for both legs. The percentage of CLP cells was similar in all groups at 2 and 4 weeks after irradiation. The same alteration tendency was observed in the 5-Gy and 10-Gy groups. There was no evident difference between the irradiated and nonirradiated legs, indicating a cell migration from one leg to the other.

A different alteration pattern was observed for the pro-B (CD19^+^ IgM^−^ C-kit^+^) cells. The percentage of pro-B cells elevated significantly in the 5-Gy and 10-Gy groups 1 week and 2 weeks after irradiation, as compared to the 0-Gy group; however, after 3 or 4 weeks, no evident changes or significant differences were exhibited in the irradiated and nonirradiated legs (Fig. 2C and 2D). The increase in the percentage of pro-B cells was dose dependent at 1 week and 2 weeks after sub-TBI.

The alteration patterns of pre-B (CD19^+^ IgM^−^ CD25^+^) cells differed between the 5-Gy and 10-Gy groups (Fig. 2E and 2F). As compared to the 0-Gy group, the 5-Gy group showed an increased percentage of pre-B cells only 4 weeks after irradiation (*P*<0.05). However, the percentage of pre-B cells significantly decreased 1 week and 2 weeks after 10-Gy sub-TBI (*P*<0.05); at 3 and 4 weeks after irradiation, the percentage of pre-B cells recovered and was significantly higher than that of the 0-Gy group. The data indicated no evident difference between irradiated and nonirradiated legs.

### The effect of radiation on the rearrangement of immunoglobulin genes

**Figure 3:**
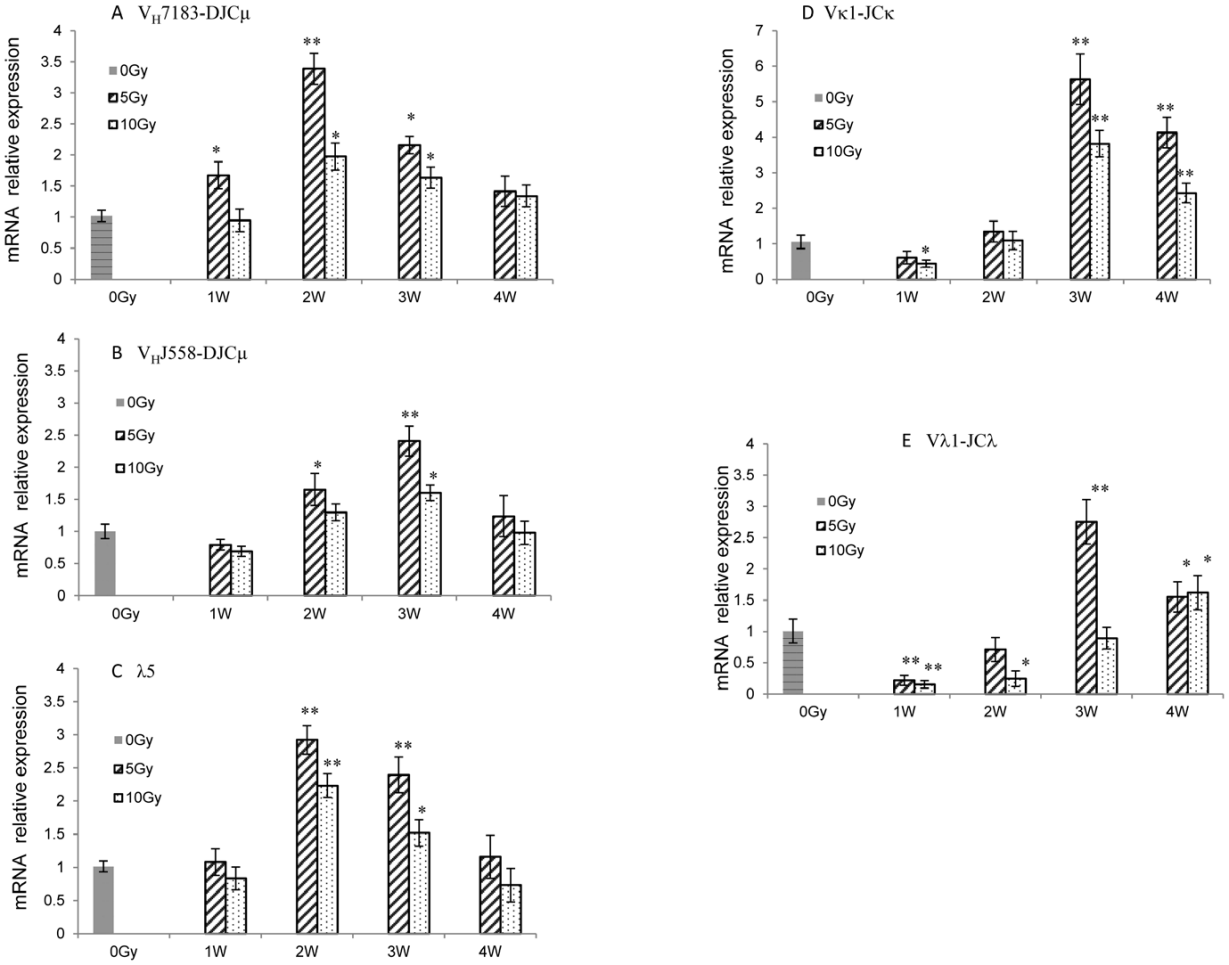
The rearrangement of immunoglobulin gene (Ig) loci in the bone marrow of irradiated mice. Real-time polymerase chain reaction analysis was performed with bone marrow cells from 6 to 8-week-old control mice (0 Gy, black bar) and sub-TBI (5 and 10 Gy) mice (gray bars). (**A**) The mRNA relative expression of Ig heavy-chain (IgH) loci V_H_7183-DJ_H_ and (**B**) V_H_J558-DJ_H_; (**C**) the pre-BCR genes surrogate light chain (SLC) component λ5; (**D**) IgL **κ** gene (**Vκ1-JCκ**); and (**E**) IgL **λ** gene (**V**λ**1-JC**λ1) rearrangements. Results are representative of 3 independent experiments. * represents *P*<0.05 and ** represents *P*<0.01, as compared with the 0-Gy group (*n* = 5).

The patterns of B-precursor cell transition can be judged by surface markers and the rearrangement of Ig genes. To further prove B-precursor cell transition patterns, IgH and IgL gene rearrangements were analyzed with RT-PCR in bone marrow cells. As shown in Figure 3A, the mRNA expression of IgH proximal genes (V_H_7183-DJ) in mouse bone marrow significantly increased 1–3 weeks after 5-Gy or 10-Gy sub-TBI. Similarly, the mRNA expression of IgH distal genes (V_H_J558-DJ) increased 2–3 weeks after sub-TBI (Fig. 3B). Proximal genes were more preferentially rearranged than distal genes in irradiated mouse bone marrow.

In addition, the mRNA expression of pre-BCR gene λ5 in mouse bone marrow increased significantly 2–3 weeks after sub-TBI (Fig. 3C). The mRNA expression of IgL κ genes (Vκ1-JCκ) and IgL λ genes (Vλ1-JCλ1) increased significantly 3–4 weeks after sub-TBI (Fig. 3D and 3E). IgL κ genes were more preferentially rearranged than **λ** genes in irradiated mouse bone marrow.

### The effect of radiation on transcription factors

**Figure 4:**
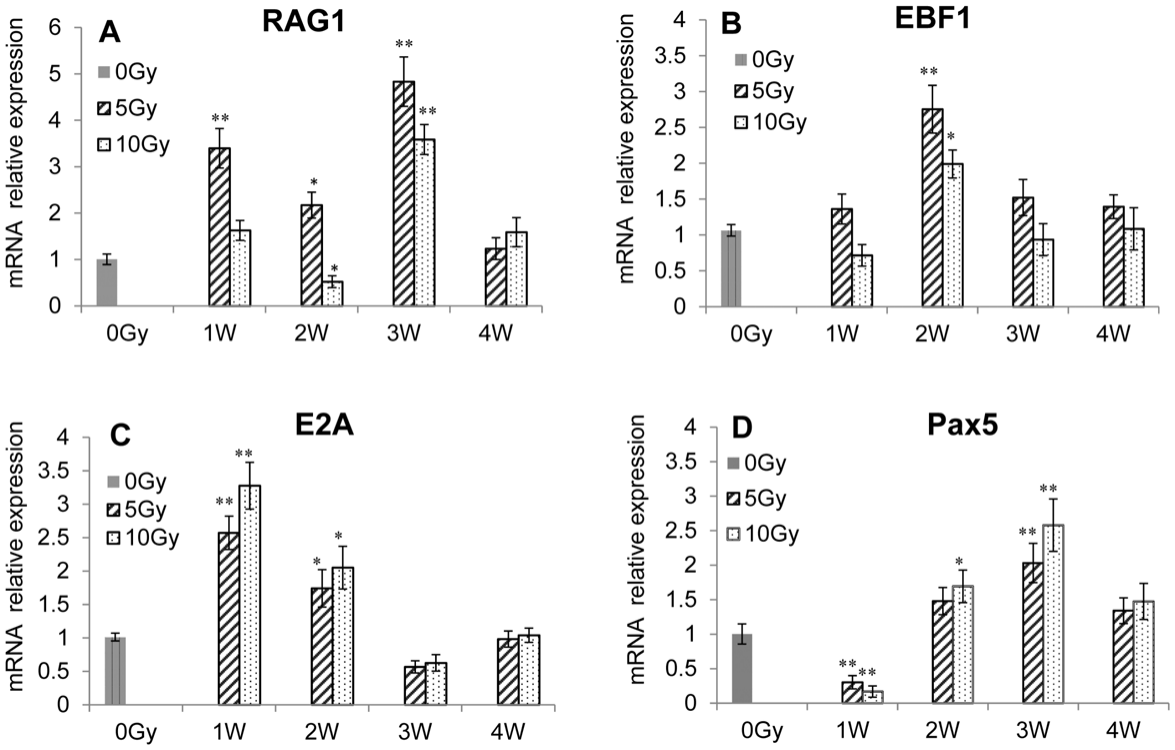
The effect of sub-TBI on transcription factors in bone marrow of irradiated mice. RT-PCR analysis of the mRNA relative expression of RAG1, E2A, Ebf1, and Pax5 in the bone marrow of 6 to 8-week-old control mice (black bar) or sub-TBI (5 and 10 Gy) mice (gray bars). Results are representative of 3 independent experiments. * represents *P*<0.05 and ** represents *P*<0.01, as compared with the 0-Gy group (*n* = 5).

The differentiation between related transcription factors was studied with RT-PCR. The Rag-1 mRNA expression increased significantly 1 week and 3 weeks after 5-Gy or 10-Gy sub-TBI (Fig. 4A). The Ebf1 and E2A mRNA expression in bone marrow increased significantly at 2 weeks (Fig. 4B and 4C). The Pax5 mRNA expression in bone marrow decreased evidently at 1 week but increased significantly at 2 and 3 weeks (Fig. 4D).

### The effect of radiation on signaling pathways

**Figure 5:**
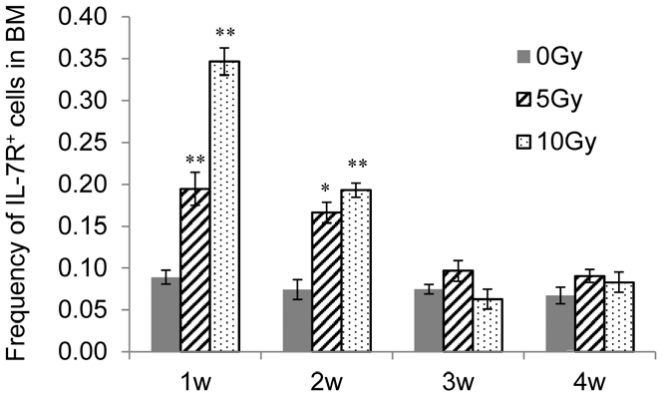
Changes in the expression of IL-7R in bone marrow cells. The percentage of IL-7R+ bone marrow cells of the control or sub-TBI (5 and 10 Gy) mice was measured with flow cytometry. Results are representative of 3 independent experiments. * represents *P*<0.05 and ** represents **P**<0.01, as compared with the 0-Gy group (*n* = 5).

The expression of IL-7R on bone marrow cells is another index for the transition of CLP cells to pro-B cells. The bone marrow cells harvested at different times after 5-Gy or 10-Gy sub-TBI were stained with IL-7R and assessed with flow cytometry. The results (Fig. 5) showed that IL-7R was significantly upregulated at 1 and 2 weeks as compared to the control, whereas no change was observed 3 or 4 weeks after irradiation.

**Figure 6:**
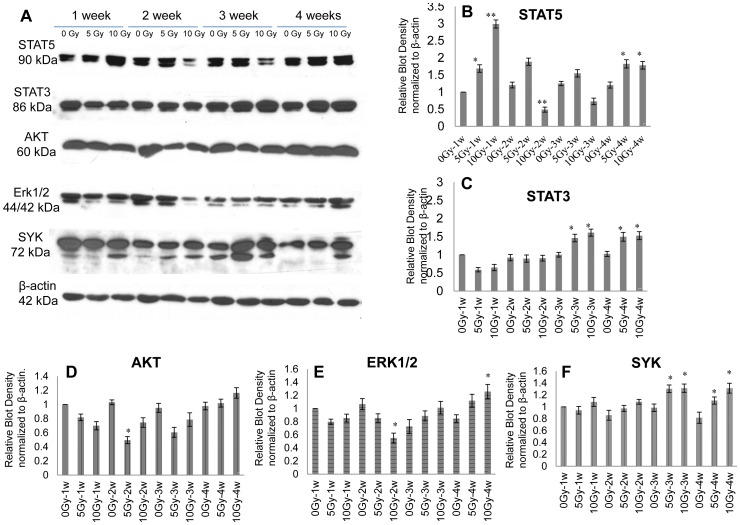
Changes in signaling proteins in bone marrow cells. The expression level of STAT5, STAT3, Akt, Erk1/2, and Syk in the bone marrow of control mice or sub-TBI mice (5 or 10 Gy) were (A) examined with Western blotting and (B) quantified with densitometer analysis. The values are presented as means ± SD of 5 mice per group, and individual values are normalized for corresponding β-actin protein levels. Results are representative of 3 independent experiments. * represents *P*<0.05 and ** represents *P*<0.01, as compared with the 0-Gy group (*n* = 5).

Other signaling pathways related to B-precursor differentiation in bone marrow were also studied with Western blotting. In the 5-Gy group, the expression of STAT5 was upregulated 1, 2, 3, and 4 weeks after irradiation. In the 10-Gy group, the expression of STAT5 upregulated significantly 1 week after irradiation, decreased at 2 and 3 weeks, and then increased again at 4 weeks (Fig. 6A, lane 1 and Fig. 6B). The expression of STAT3 decreased 1 week after irradiation but upregulated significantly at 3 and 4 weeks (Fig. 6A, lane 2 and Fig. 6C). The expression of Akt and Erk1/2 decreased 1 and 2 weeks after sub-TBI but gradually upregulated at 3 and 4 weeks, as compared to the control group (Fig. 6A, lane 3 and 4, Fig. 6D and 6E). The expression of SYK decreased 1 and 2 weeks after sub-TBI but gradually upregulated at 3 and 4 weeks (Fig. 6A, lanes 4 and Fig. 6F).

## Discussion

This study sought to explore the impact of ionizing radiation on the early stage of B-cell development via an examination of the transition of CLP to pro-B to pre-B cells within bone marrow as a function of radiation doses and times in a sub-TBI mouse model in which one leg of the NIH Swiss male mouse was shielded from the radiation field to avoid bone marrow failure during the study period. The results demonstrated that: (1) there was a significant radiation dose-related reduction in the numbers of bone marrow stem cells, including CLP, pro-B, and pre-B cells (Fig. 1); (2) the preprogrammed transition of CLP to pro-B to pre-B cells was continuously processed after sub-TBI, as evidenced by the shift of surface lineage markers and the sequential rearrangement of Ig genes (Figs. 2 and 3), leading to a gradual recovery of the B-lymphocyte population for maintaining humoral immunity; and (3) the transition of CLP to pro-B to pre-B cells within bone marrow was related to transcription factors of RAG-1, E2A, EBF1, and PAX5 (Fig. 4) and the signaling pathways of IL-7R, STAT3, STAT5, AKT, Erk1/2, and SYK (Figs. 5 and 6).

Although high-dose irradiation (5–10 Gy) to large volumes of bone marrow, such as with one leg shielded (sub-TBI), does not completely suppress bone marrow regeneration, it does result in a rapid compensatory response [17]. Rebound responses that occurred in shielded extremities were similar to those seen in radiation-depleted femurs (Fig. 2). This regeneration, which allows for the repopulation of irradiated or damaged areas, may be attributed to hematopoietic stem cell or progenitor cell mobilization from nonirradiated areas of bone marrow that is followed by circulatory trafficking [18], [19]. The elevation of cytokine levels could also be partly responsible.

We found that the transition of B-precursor cells after mouse bone marrow sub-TBI was a highly ordered process. First, in the cell transition, the percentage of CLP cells was markedly upregulated 1 week after irradiation. The percentage of pro-B cells significantly increased 1–2 weeks after irradiation, followed by an increased percentage of pre-B cells within 3–4 weeks after irradiation (Fig. 2). Consistent with other reports, we found that this pattern was the same in both irradiated and nonirradiated legs [17], [18], [19]. Second, the sequential rearrangement of Ig genes in the B-precursor transition in bone marrow after sub-TBI was also a highly ordered process, including upregulated mRNA expression of IgH proximal genes (V_H_7183-DJCμ) and distal genes (V_H_J558-DJCμ) in the pro-B cell stage at 2–3 weeks after irradiation. Proximal genes were rearranged more preferentially than distal genes in the bone marrow of irradiated mice. Subsequently, Igκ and Igλ variable regions in the pre-B cell stage were assembled at 3–4 weeks after irradiation. IgL κ genes were more preferentially rearranged than λ genes in the bone marrow of irradiated mice (Fig. 3).

Differentiation of the CLP to committed pro-B cells depends on the 3 transcription factors: E2A, EBF, and Pax5 [20]. All 3 factors are also essential for V(D)J recombination of the IgH gene during early B-cell development. The B-precursor transition in bone marrow after sub-TBI at 5 or 10 Gy is likely to involve these transcription factors, as evidenced by the increased expressions of RAG-1, E2A, EBF1, and PAX5 at 1–2 weeks (Fig. 4); this is consistent with the time point of the IgH locus rearrangement at the CLP or pro-B cell stage (Fig. 3). In addition, RAG-1 and PAX5 gene expressions are significantly increased at 3 weeks after sub-TBI, which is consistent with the time point of the IgL locus rearrangement at the pre-B cell stage (Fig. 3).

IL-7R activity is both necessary and sufficient for CLP to differentiate into early B-lineage precursors. IL-7 activates 3 major signaling pathways: the JAK-STAT, the phosphatidylinositol 3-kinase (PI3K)–Akt, and the Ras-Raf-Erk [21], [22]. IL-7 signaling is also required for the developmental transition from pro-B to pre-B cells, where it acts in synergy with pre-BCR signaling to promote the expansion of large pre-B cells bearing in-frame IgH rearrangements [10], [23], [24], [25]. Our results showed that the expressions of IL-7R and STAT5 in bone marrow cells increased significantly 1 week after sub-TBI (Fig. 5 and Fig. 6, lane 1). The data demonstrate that IL-7R/STAT5 signaling is critical for the development of CLP into pro-B cells after irradiation. In addition, STAT3, STAT5, and Erk1/2 protein expression in bone marrow cells increased significantly 3 and 4 weeks after sub-TBI (Fig. 6, lane 1–4), which reflects their important roles in pre-B cell regeneration [26], [27].

Studies have shown that SYK has a central role in the activation of pathways that regulate the proliferation and differentiation of pre-B cells. SYK is also involved in facilitating IgL chain gene recombination and subsequent differentiation of pre-B cells to immature B cells [16], [28]. Our results showed that the expression of SYK expression in bone marrow cells increased significantly 3 and 4 weeks after sub-TBI, indicating that SYK also plays a critical role in pre-B cell regeneration after irradiation (Fig. 6, lane 5).

The data indicate that, although certain doses of radiation damage lymphocytes, they also promote the highly ordered differentiation of the lymphoid system in the bone marrow. One week after sub-TBI is the key time point for the transition from CLP to pro-B cells. Two weeks after sub-TBI is not only the key time point for the transition from the pro-B cells to pre-B cells but also the main checkpoint for Ig gene rearrangement. These transition patterns may reflect the functional processes that are preserved in the residual radioresistant cells to restore the immunity that has been greatly damaged by radiation.
